# SOCIAL ISOLATION OF OLDER ADULTS IN A MARGINALIZED COMMUNITY IN JAPAN

**DOI:** 10.1093/geroni/igae098.1889

**Published:** 2024-12-31

**Authors:** Yuichi Watanabe

**Affiliations:** Musashino University, Nishitokyo, Tokyo, Japan

## Abstract

Japan stands as one of the most aged societies globally, with approximately 29% of its population aged 65 or older as of 2022. Previous studies indicate that 10-30% of Japanese older adults experience social isolation, with 2-10% facing severe social isolation (Saito, 2009). This issue is particularly prevalent among those who have contact with others less than once a week, posing potential health risk (Saito et al., 2015). However, it’s crucial to acknowledge the existence of extremely aging communities in Japan, especially in rural areas, known as marginalized communities, where over 50% of the population is 65 years or older. This presentation offers insights obtained from surveys conducted in 2015, 2017, and 2019 within one such marginalized community. The findings reveal that out of 297 older adults surveyed, 72 were living alone. Among them, 4 reported meeting others less than once a week, while 18 met others less than four times a week. These results suggest that approximately 25% of older adults living alone may experience social isolation, with 5.6% facing severe social isolation. While these findings may not be generalizable to all marginalized communities in Japan, they highlight the heightened risk of social isolation within such contexts. Future research should conduct detailed analyses to inform discussions and develop solutions for addressing social isolation in marginalized communities.

